# Style in Medical Authorship

**Published:** 1910-12-31

**Authors:** 


### The Hospital

A Journal of

The Medical Sciences and Hospital Administration.

New Series. No. 204, Vol. VIII. [No. 1276, Vol. XLIX.] SATURDAY, DECEMBER 31. 1910.

### Style in Medical Authorship.

Of artists generally, but particularly of writers, it has been well said that the style is the man. There must be exceptions to this rule, for surely modern medical literary style cannot represent the modern medical man. No one can fail to see that in every application of the term our profession is a rising one. Molière and the encyclopædists laughed at it with some reason, it is true, and for a while advance was slow. But then there came, within a period of forty years, the discoveries of chloroform, antiseptic surgery, and the new applied science, bacteriology; and the work of living minor novelists contains a reflection of the altering attitude of society in an amusingly complete and sudden change of the tone they began with, a tone taken from Thackeray with his " Sam Huxter of Bart's." Intellectual leaders now find themselves under the necessity of getting up a little medical science, and not neglecting the copy of the *Lancet* which has found its way on to the library table of their club. But still the doctor in the ward, or the operating theatre, or the laboratory, or the town hall public health office, is a very different person from what you would imagine him to be from his appearances in print. At work, he is a rather unusual combination of the man of action and the thinker—more of a thinker than the barrister or the diplomat, more practical than the scholar or the pure scientist, and owing on the whole but little, it is pleasant to think, to social connections, and nothing to privilege. But as an author, he exemplifies all the faults which Mr. Asquith noted in a recent able rectorial address at Aberdeen. For instance, it is the commonest thing for him to take four thousand words to express badly what could be neatly put in four hundred. Worse still, he has a literary failing almost peculiar to himself, a failing for which no less, and no less remote, a person than Sir Thomas Browne is responsible.

Sir Thomas Browne, as all the critics are agreed, was a virtuoso in the use of the English language. He tossed words about, says Mr. Edmund Gosse, in a rather mad sort of way. But then he had on his side genius, and artistic selection, and withal rare thoughts and fancies to deliver himself of. And he could write simply too, as in the sentence: " The hunters are up in America," which aroused Coleridge's admiring wonder. Now, most unfortunately, just because Sir Thomas Browne was a doctor of medicine, and a man to be proud of, and a man to admire whom is a sign of taste, it has become the fashion and the custom for sundry eminent medical men to mistake the literary novice's tendency to high-flown diction for a genuine calling to write like Sir Thomas Browne; and to yield to the fatal impulse most complacently. This bad lead lesser men have followed, and these lesser again; in the result, a great deal of verbosity, redundance, " deliberate picturesqueness "—certainly no second master of English prose from the ranks of the medical profession. It is therefore of interest to see if the Prime Minister's suggested remedies can have any application in the case under notice, the case of medical literature and debate.

What Mr. Asquith desiderated was for a university to make it its business expressly to teach style; that is, the proper manner of presenting one's matter. Of course he meant style not solely in the artistic sense of the word but in its wider connotation. No one by taking thought or pains could come to have at command a poet's liquid prose, but it should be simple for any sensible person to submit to the process of having the elements of literary craftsmanship drilled into him. The ordinary business man is a case in point. His letters may not be models of composition, and they display the strongest attachment to conventionality in the matter of verbiage; but, on the other hand, it is impossible to mistake their meaning, just because such mistakes would be inconvenient and possibly costly; and (again on the score of practical disadvantage) they waste no space or anybody's time. Would that the same could be said of the ordinary medical article, or brochure, or text-book! In France, perhaps, this is the case, for the French language lends itself rarely to lucidity and elegance. But in this country we have painfully few who, like Professor Osler, Mr. Havelock Ellis, and Dr. Bulloch, can produce a page of workmanlike prose. The lack, if not supplied, must be felt more and more as time goes on, for with the rapid forward march of medicine its literature perforce increases at a rate which dismays one who looks into the future. The amount of knowledge to be taken note of by leaders of the profession fifty years hence will be colossal. Before then, let us hope, such knowledge will be recorded by trained writers only, and if a medical author cannot manage to express himself with strength and clearness—the first two requisites of style—he will be wise enough to collaborate. Even now, one very favourable review, at all events, awaits the medical text-book which is avowedly a recension by a competent lay journalist of a draft manuscript handed to him by a well qualified medical author. It is sad to think of the library storage-space which might thus have been saved in the last twenty or thirty years. But there are naturally some objections to such a plan, and the only alternative would appear to be individual post-graduate instruction for intending authors in the neglected art of English composition as applied to the practical needs of to-day. As Mr. Asquith has hinted, our universities do not do their duty in this respect.

